# 18 Oral Ketamine for Pain Management During Bromelain Based Enzymatic Debridement After Burn Injury

**DOI:** 10.1093/jbcr/iraf019.018

**Published:** 2025-04-01

**Authors:** Deanna DeHoff, Elizabeth Halicki, Courtney Tresslar, Melanie Condeni, Jason Hirsch, Adria Johnson, Steven Kahn

**Affiliations:** South Carolina Burn Center at MUSC; Urgo Medical; Medical University of South Carolina; South Carolina Burn Center at MUSC; Medical University of South Carolina; Medical University of South Carolina; Medical University of South Carolina

## Abstract

**Introduction:**

Bromelain based enzymatic debridement in burn injuries is now commercially available in the US. Literature suggests multiple benefits, but the enzyme is painful. Some centers have utilized moderate/deep sedation, regional blocks, or continuous infusions of sedatives in the intensive care unit, but these methods require increased resources and monitoring. Oral ketamine has been shown to be a safe, simple alternative for burn patients and has been utilized for bromelain. The authors hypothesize oral ketamine attenuates the pain increases seen during bromelain bromelain based enzymatic debridement.

**Methods:**

This was a single institution retrospective review of patients who were premedicated with oral ketamine (1-4 mg/kg 30 to 45 minutes prior to bromelain application). Patients also received their personalized baseline oral multimodal pain regimen and oral lorazepam prior to the procedure. Data collection included demographics, burn characteristics, concomitant analgesic meds, pain scores, vital sign monitoring, and length of stay (LOS). A Wilcoxon Signed-Rank test was used to compare baseline pain with pain after bromelain was applied- and after it was removed Pain scores were scored verbally 0-10; patients observed as sleeping or reporting minimal pain were defined as a pain score of 0. Severe pain defined as >7, moderate was 5-7, minimal pain was 1-4. Patient vitals were continuously monitored, and oxygen/capnography was used during the procedure. Patient were not made NPO.

**Results:**

Twenty-three patients were included. Median age was 31 years IQR [22,44], with 17 males and 5 females. Burn size was 6%TBSA [IQR4,15]. Median ketamine dose was 224.5 mg IQR [172.5,280]. During the 8-hour period, the median morphine milligram equivalents were 30, IQR [15,40] and the median lorazepam milligram equivalents) were 2, IQR [1,3]. The median baseline, pre-treatment pain score was 3.25/10; during the 4-hour treatment window was 4.5, IQR [0,8], p=0.258. and the post treatment pain score was 5. Pain scores did not increase significantly with bromelain + oral ketamine. in pain. Eight patients were dosed with labetalol for hypertension; and no adverse events were noted. The median LOS was 12 days.

**Conclusions:**

This data shows pain is well controlled at a moderate level post bromelain application and does not significantly increase from baseline with administration of oral ketamine.

**Applicability of Research to Practice:**

Moderate, deep, and continuous sedation was avoided and limited amounts of opioids were used. The use of oral ketamine prevented additional NPO time. Additional studies are necessary to further optimize this practice.

**Funding for the Study:**

N/A

